# Exposure to Nepalese Propolis Alters the Metabolic State of *Mycobacterium tuberculosis*

**DOI:** 10.3389/fmicb.2022.929476

**Published:** 2022-06-23

**Authors:** Rafał Sawicki, Jarosław Widelski, Piotr Okińczyc, Wiesław Truszkiewicz, Joanna Glous, Elwira Sieniawska

**Affiliations:** ^1^Chair and Department of Biochemistry and Biotechnology, Medical University of Lublin, Lublin, Poland; ^2^Department of Pharmacognosy, Medical University of Lublin, Lublin, Poland; ^3^Department of Pharmacognosy and Herbal Medicines, Wroclaw Medical University, Wrocław, Poland; ^4^Department of Natural Products Chemistry, Medical University of Lublin, Lublin, Poland

**Keywords:** flavonoids, metabolomics, LC-MS, sigma factors, oxidative stress, natural products

## Abstract

Propolis is a natural product proved to be efficient against *Mycobacterium tuberculosis*. Although it is produced by bees, its active alcoholic-aqueous fraction contains plant-derived molecules. To gain some insight into its mechanism of antimycobacterial activity, we studied the metabolic changes in bacterial cells treated with extract of *Trigona* sp. propolis from Nepal. The detailed metabolomic and transcriptomic analysis performed in this study indicated target points in bacterial cells under propolis extract influence. The profile of lipids forming the outer and middle layer of the mycobacterial cell envelope was not changed by propolis treatment, however, fluctuations in the profiles of amphipathic glycerophospholipids were observed. The enrichment analysis revealed bacterial metabolic pathways affected by *Trigona* sp. propolis treatment. The early metabolic response involved much more pathways than observed after 48 h of incubation, however, the highest enrichment ratio was observed after 48 h, indicating the long-lasting influence of propolis. The early bacterial response was related to the increased demand for energy and upregulation of molecules involved in the formation of the cell membrane. The transcriptomic analysis confirmed that bacteria also suffered from oxidative stress, which was more pronounced on the second day of exposure. This was the first attempt to explain the action of Nepalese propolis extract against mycobacteria.

## Introduction

Natural resources have been explored for their antimicrobial activity for decades (Newman and Cragg, 2020). Many compounds originating from plants, bacteria, or fungi have found application in the treatment of infectious diseases (Newman and Cragg, 2016). The screening for antimycobacterial molecules is ongoing and results in the discovery of some potent secondary metabolites (Salomon and Schmidt, 2012; Chinsembu, 2016). Propolis can be listed among them. It is a honey bee product, formed by mixing bee saliva, beeswax and plant resins, and essential oils of different botanical sources (Simone-Finstrom and Spivak, 2010). Most of its biochemically active components originate from plant resins and their phytochemical composition is crucial for propolis biological properties (Simone-Finstrom and Spivak, 2010; Isidorov et al., 2016). Nepalese propolis is a subtype of “red propolis” (Okińczyc et al., 2020). Its main components are flavonoids (mainly aglycones of isoflavones, neoflavanoids, flavanones, and some chalcones), pterocarpans, and some specific quinones (Awale et al., 2005; Shrestha et al., 2007; Okińczyc et al., 2020). The phytochemical analysis performed for samples used in this study revealed that the main chemical constituents of studied extracts were 2'-hydroxyformononetin, odoratin, vestitol, butein, dalbergin, 7-hydroxyflavanone, and pinocembrin (Okińczyc et al., 2020).

The early reported antimycobacterial activity of propolis came from 1990, when Grange and Davey found that 1:320 dilution of propolis ethanolic extract inhibited the growth of *Mycobacterium tuberculosis* H37Rv reference strain (Grange and Davey, 1990). Later, Scheller et al. discovered synergistic effects of the propolis ethanolic extract and antitubercular drugs against isolated clinical mycobacterial strains (Scheller et al., 1999). The possible mechanism of general antibacterial activity of propolis was related to the action of its individual polyphenolic constituents of plant origin (Cushnie and Lamb, 2005; Almuhayawi, 2020). It was associated with: cytoplasmic membrane function alteration, nucleic acid synthesis inhibition, energy metabolism inhibition, or the reduction of biofilm formation (Cushnie and Lamb, 2005; Almuhayawi, 2020). Notably, most of the reports focused on poplar propolis's antibacterial mechanism of action. Rest types of propolis were not investigated so vast. In addition, no specific information can be found about the propolis's influence on the metabolism of *M. tuberculosis*.

Therefore, in this work, we aimed to determine antimycobacterial activity of the 70% ethanolic extract prepared from Nepalese Apis mellifera L. and Trigona sp. propolis samples. Later, we exposed mycobacteria to more potent Trigona sp. propolis for 24 and 48 h and analyzed the changes in bacterial metabolites and in the transcription of genes coding bacterial regulatory network. This was the first attempt to explain the action of Nepalese propolis extract against mycobacteria.

## Materials and Methods

### Preparation and Characterization of the Propolis Extract

Both types of propolis (*Apis mellifera* L. propolis and *Trigona* sp. propolis) were collected from apiaries in Korak village (27.66°N 84.69°E) in Chitwan District in Nepal. The 70% ethanolic extract was prepared and phytochemically characterized according to the previously described procedure (Okińczyc et al., 2020). Briefly, 5 g of each grounded propolis sample was extracted with 50 ml of 70% ethanol in an ultrasonic bath (40°C for 45 min and 756 W). The obtained extracts were stored at room temperature for 12 h and then filtered through a filter paper (Whatman No. 10). Then, ethanol was removed from the extracts under reduced pressure, and the samples were frozen and lyophilized. The extraction yielded 1.34 g of the extract from *Apis mellifera* propolis and 1.76 g of the extract from *Trigona* sp. propolis.

### Tested Organism and Culture Conditions

#### Inoculum Preparation

The inoculum of *M. tuberculosis* H37Ra (ATCC25177) was prepared as described previously (Sawicki et al., 2018). Briefly, bacteria were grown on the Löwenstein–Jensen slopes (BioMaxima, Lublin, Poland) for 2 weeks, and transferred to 5 ml of the fresh Middlebrook 7H9 broth supplemented with 10% ADC and 0.2% glycerol (MilliporeSigma, St Louis, USA). After 3 min vortexing with 1 mm glass beads, the bacteria were left for sedimentation at room temperature (30 min). The upper 2 ml was transferred to a new sterile tube and left for sedimentation (15 min). Finally, upper 1 ml of supernatant was transferred in a sterile tube and turbidity was adjusted to 0.5 McFarland standard (1E+08 CFU/ml) with ADC supplemented Middlebrook 7H9 broth. That standardised bacterial suspension was diluted 100 times with ADC supplemented Middlebrook 7H9 broth to a final density of 1E+06 CFU/ml.

#### Minimal Inhibitory Concentration

Minimum inhibitory concentration (MIC) was performed according to CLSI guideline (Woods et al., 2011) as described previously (Sawicki et al., 2018). Propolis was tested in the concentration range from 256 to 2 μg/ml. In short: first the ethanol-free, dry propolis extract was dissolved in dimethyl sulphoxide (DMSO; MilliporeSigma, St Louis, USA), then the serial 2-fold dilutions in the 7H9-S medium were prepared. The final DMSO concentration did not exceed 2% (v/v). Isoniazid, ethambutol, rifampicin, and streptomycin (MilliporeSigma, St Louis, USA) were used as reference standards in a concentration range from 0.001 to 16 μg/ml. The round-bottom 96 microwell plates included test wells (filled with 50 μl of inoculum and 50 μl of extract dilutions) and control wells (sterility, growth, and 2% DMSO controls), which were sealed with adhesive foil to prevent liquid evaporation and incubated for 8 days at 37°C. After the incubation period, 10 μl of resazurin (AlamarBlue, Invitrogen, Carlsbad, CA, USA) solution was added to all wells and placed again at 37°C for 48 h. The MIC was defined as the lowest extract concentration preventing blue to pink change in test wells. The determination of MIC values was performed in duplicate.

#### Bacteria Exposure to Propolis Extract

Four high-density cultures were propagated, each of 400 ml culture medium. The 4 ml of initial *M. tuberculosis* H37Ra suspension (inoculum) has been added to each flask with 400 ml of culture medium and incubated at 37°C with aeration of 100 rpm for approximately 4 weeks. This yielded a high-density culture of around 1 × 10^9^ CFU/ml (Sieniawska et al., 2021). The experimental culture was supplemented with *Trigona* sp. propolis extract dissolved in DMSO (final concentration 256 μg/ml normalized by weight), while the control culture was supplemented with 2% DMSO. Two flasks (test and control culture) were incubated for 24 h and additional two flasks for 48 h. Then, the bacterial metabolism was stopped by adding cold methanol (−60°C) (1:1v/v) to each culture. Next, the cultures were aliquoted in 50 ml Falcone tubes and centrifuged for 30 min at 8,000 rpm at 4°C. Bacterial pellets (each from 50 ml of culture) were rinsed three times with cold phosphate-buffered saline (Biomed, Lublin, Poland) and centrifuged again to remove traces of medium. Prior to analysis, the bacterial biomass was lyophilized, weighed, and stored at −60°C before analysis.

### Gene Expression Analysis

#### Total RNA Extraction

After 24 h incubation and before the metabolite quenching, 2 ml of the culture exposed to propolis extract and the control culture were collected and centrifuged. The bacterial pallet was resuspended in 1 ml of RNA pro solution (MP Biomedi-calsaterials, Santa Ana, USA) and placed in a tube with 0.8 ml zirconia beads (0.1 mm diameter). Cells were agitated in a bead-beater (FastPrep24 instrument, MP Biomedicals, Santa Ana, USA MP Biomaterials) at the highest speed by two 45 s pulses. Remains were centrifuged, and the liquid was transferred to a new tube. Total RNA was isolated to the manufacturer's directions with FastRNA Pro Blue Kit (MP Biomedicals, Santa Ana, USA, MP Biomaterials). The RNA concentration and purity were measured spectrophotometrically, then aliquoted and stored at −80°C for future use.

#### qPCR Reactions

qPCR reactions were performed with LightCycler®EvoScript RNA SYBR®Green I Master kit (Roche, Basel, Switzerland) in LightCycler®480 thermal cycler (Roche, Basel, Switzerland). The total volume per reaction was 20 μl, and the composition was as follow master 5 × 4 μl, primer mix 20 × (10 μM) 1 μl, RNA template 5 μl (1 ng), and water 10 μl. Cycling conditions: reverse transcription 60°C for 15 min, initial denaturation 95°C for 10 min, 40 cycles of amplification 95°C for 10 s followed by 30 s incubation at 58°C and melting curve from 25 to 95°C with ramping rate 2.2°C/s. For relative quantification of transcripts, targets were normalized to 16S rRNA. Primers (Millipore, Sigma-Aldrich) are listed in Table 1. Relative mRNA quantification was calculated according to the delta–delta mathematical model.

**Table 1:** Primers used for qPCR analyses.

**Gene**	**Primer pair (5^**′**^-3^**′**^)**
*sigA:*	FOR: GACGAAGACCACGAAGAC REV: TCATCCCAGACGAAATCAC
*sigB:*	FOR: CTCGTGCGCGTCTATCTGAA REV:AGCAGATGCTCGGCATACAA
*sigE:*	FOR: AACCCCGAGCAGATCTACCA REV: CTCGATGTCACACAGCACCA
*sigG:*	FOR: CGTCAATGAGCCTACGCAGA REV:GCGAAATTCCGTTCAGTCCG
*sigH:*	FOR: GCCGCTGTTTCTTGCGATAG REV: CCAGGAGACGATGGTGAAGG
*sigM:*	FOR:CGTCAGCAGTTGGTTGCAC REV: ACATCTTCTAGAGGGGCGGT
*sigL:*	FOR: CGTGATCCAGCGGTCCTAC REV:CAATCGCGACTTCACCGTTC
*sigJ:*	FOR:GACCAGCCCGAGTATGAACC REV:ATCCCGACGTGACGTTTACC
*sigF:*	FOR:CCGCAGATGCAGTTCCTTGA REV: GGTCGGACTTCGTCTCCTTC
*sigD:*	FOR: AACAATCTCGTCCTTCAGCCG REV:CGAGATCCTCATTCTGCGTGT
*sigK:*	FOR: CCGCCACACCTCAAGATAGA REV: TCTACGACCACACCAAGTCG
*sigC:*	FOR: TTACCGCACTCGCCTTGTC REV: GGACAGATAGGCGACGAACC
*sigI:*	FOR:AAGACATGGTGCAAGAGGCA REV: ACGCCGACTTGATGTGATCC
*16S RNA*	FOR: ACTTCGGGATAAGCCTGGGA REV: AGCGCTTTCCACCACAAGAC

### Bacterial Metabolites Extraction and Analysis

#### Metabolites Extraction

To extract metabolites present in the bacterial biomass, around 30 mg of lyophilized bacteria per sample (10 samples) were weighted, poured with mixture of chloroform, methanol, and water (2:1:0.1; v/v/v; 1.5 ml; lipids extraction) or another 30 mg (10 samples) poured with mixture of methanol and water (1:1; v/v; 1.5 ml; more polar compounds) and sonicated for 20 min. After centrifugation (10 min at 13,000 rpm at 4°C) the supernatant was collected, while bacterial residue was extracted again. Combined supernatants were evaporated to dryness under reduced pressure at 30°C and stored at −20°C before analysis.

#### Bacterial Metabolites Analysis

Extracted metabolites were dissolved in acetonitrile–methanol–isopropanol (1:1:2 v/v/v) and filtered through 0.22 μm PTFE syringe filters. The chromatographic and mass spectrometry conditions were as described previously (Sieniawska et al., 2021). Metabolites were separated on Gemini® chromatographic column (3 μm i.d. C18 with TMS end-capping, 110 Å, 100 × 2 mm) supported by a guard column (Phenomenex Inc, Torrance, CA, USA), using Agilent 1200 Infinity HPLC chromatograph (Agilent Technologies, Santa Clara, CA, USA). The solvents were pumped at the flow rate of 0.3 ml/min to perform the gradient of phase A [water with 0.1% formic acid (v/v)] and phase B [0.1% formic acid in acetonitrile (v/v)]: 5 min, 0% B; 20 min, 66% B; 35 min, 95% B. Then, metabolites were detected on Agilent 6530B QTOF spectrometer equipped with a Dual Agilent Jet Stream spray source (ESI) (Agilent Technologies, Santa Clara, CA, USA). The operating conditions were as follows: sheath gas temp: 400°C, sheath gas flow: 12 L/min; drying gas temp: 350°C, drying gas flow: 12 L/min; nebulizer pressure: 40 psig, capillary V (+): 4000 V, skimmer 65 V. The acquisition was performed in a positive ion mode taking 2 spectra/s in a scan range from 100 to 3,000 *m/z*.

#### Bioinformatic Analysis

Raw files were converted to mzDATA format using Mass Hunter Qualitative Analysis software (version B.07.00; Agilent Technologies, Santa Clara, CA, USA). Features were extracted in the open–source software XCMS (version Version 3.7.1, https://xcmsonline.scripps.edu) applying centWave algorithm. The correction of retention time was done in obiwarp method. After normalization, scaling, and filtering the pairwise statistical analysis was performed. Features were retained only if present in at least 5 replicates at the intensity threshold of 500. The molecular features present in propolis extract were removed from test samples. The differences between test and control groups were statistically significant at *p* < 0.05 (Welch *t*-test). Only statistically significant features were annotated and further analyzed. Metlin database (https://metlin.scripps.edu) and MS–LAMP software (http://ms-lamp.igib.res.in) were used to tentatively identify the dysregulated molecules. Enrichment analysis was performed in MetaboAnalyst 5.0 (https://www.metaboanalyst.ca).

## Results

### Minimal Inhibitory Concentration of 70% Ethanolic Extracts of Propolis

Minimum inhibitory concentration values of Nepalese propolis extracts, as well as antibiotics, were presented in Table 2. Both extracts exhibited bacteriostatic activity against *M. tuberculosis* H37Ra. However, the inhibitory activity of propolis obtained from Trigona sp. was higher than *Apis mellifera* L. propolis. Therefore, *Trigona* sp. propolis extract was used in further metabolomic research.

**Table 2:** MIC values of propolis extract and antibiotics against *M. tuberculosis* H37Ra.

**Sample**	**MIC [μg/mL]**	Reference^*^MIC [μg/mL]
*Apis mellifera* L. propolis extract	32	
*Trigona* sp. propolis extract	8	
Ethambutol	2	2
Streptomycin	2	0.5
Isoniazid	0.25	0.25
Rifampicin	0.004	0.008

* *Heinrichs et al. (2018)*.

### Bacterial Metabolites Analysis

**Figure 1:**
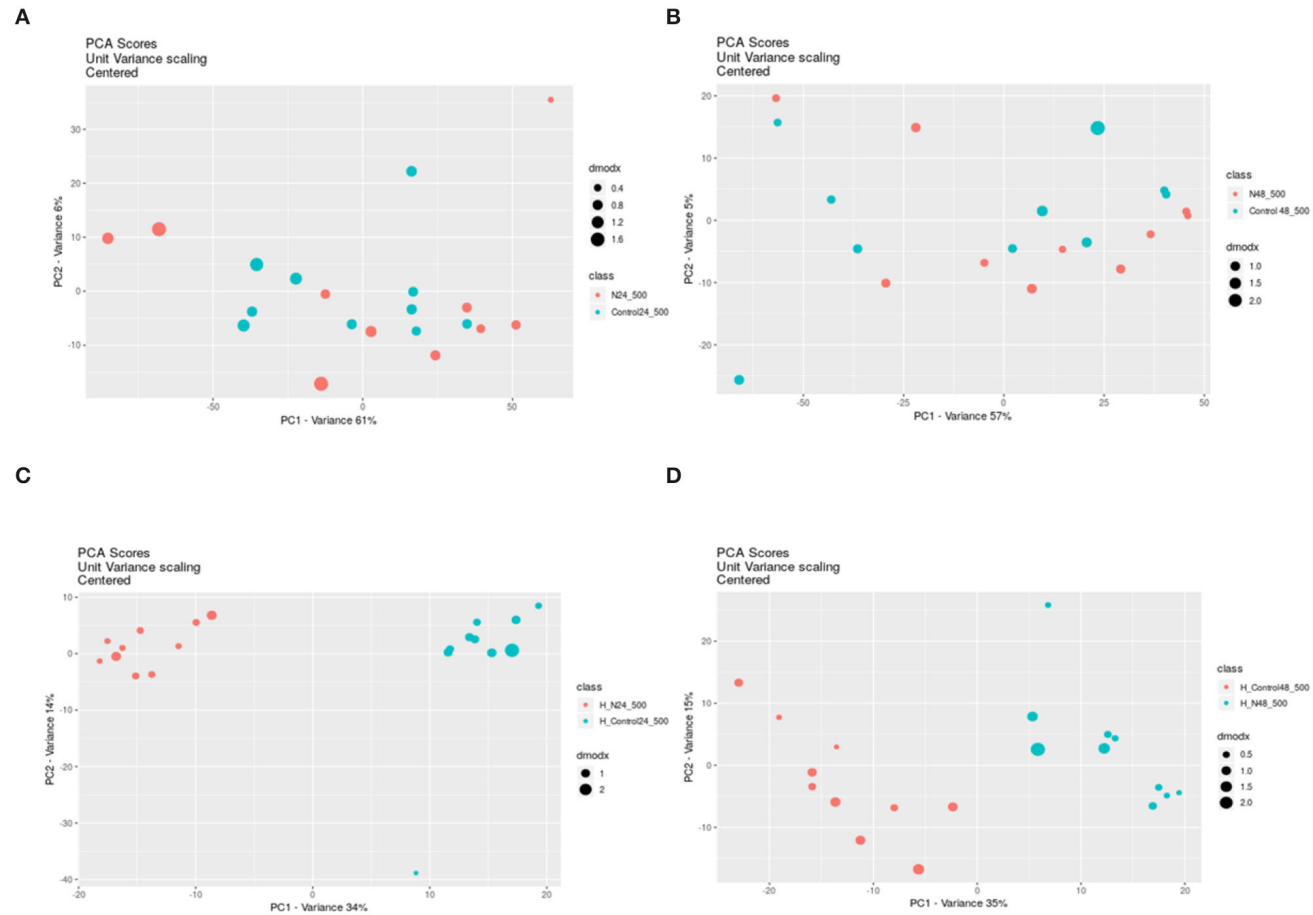
The PCA scores from pairwise analysis (XC–MS online) of samples obtained from control and treated bacteria extracted with different solvents and after different times. PCA was applied to decrease high dimensionality of metabolomic data by the reduction of high-dimensions data into lower dimensions and retention of as much information as possible. PCA shows if any separation between groups of samples exists. Chloroform-methanolic extracts after 24 h exposure **(A)** and after 48 h exposure **(B)**; Methanolic–aqueous extracts after 24 h exposure **(C)** and after 48 h exposure **(D)**; N – propolis treated bacteria (blue dots), Control–control bacteria (red dots). DmodX is the distance between the original data point and the model plane in X-space, it identifies the outliers.

**Figure 2:**
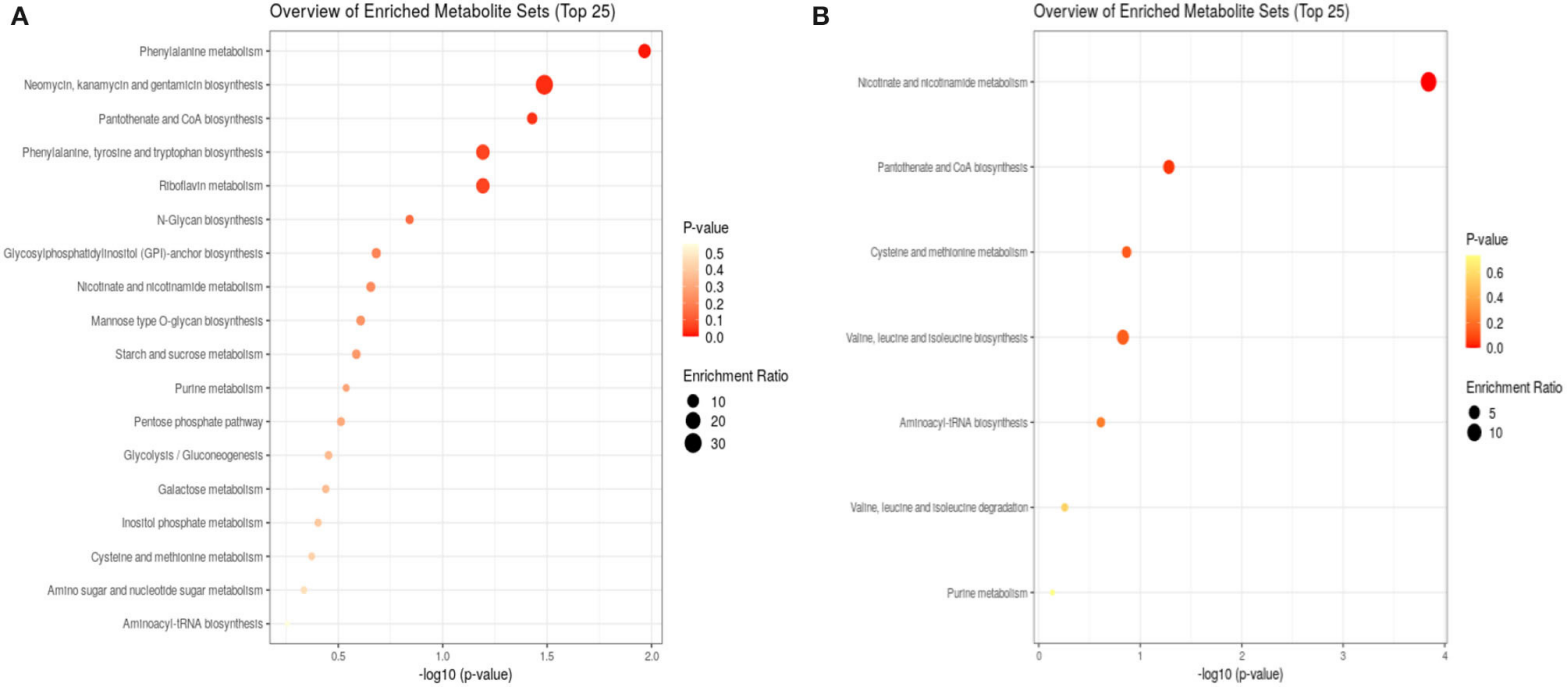
Enrichment analysis of bacterial metabolites after 24 h **(A)** and 48 h **(B)** exposure to propolis. After 24 h, eighteen metabolite sets were enriched, while after 48 h only seven sets. The size of dots represents the enrichment ratio, which is calculated as the number of hits within a particular metabolic pathway divided by the expected number of hits. The color intensity corresponds to the *p*-value.

**Figure 3:**
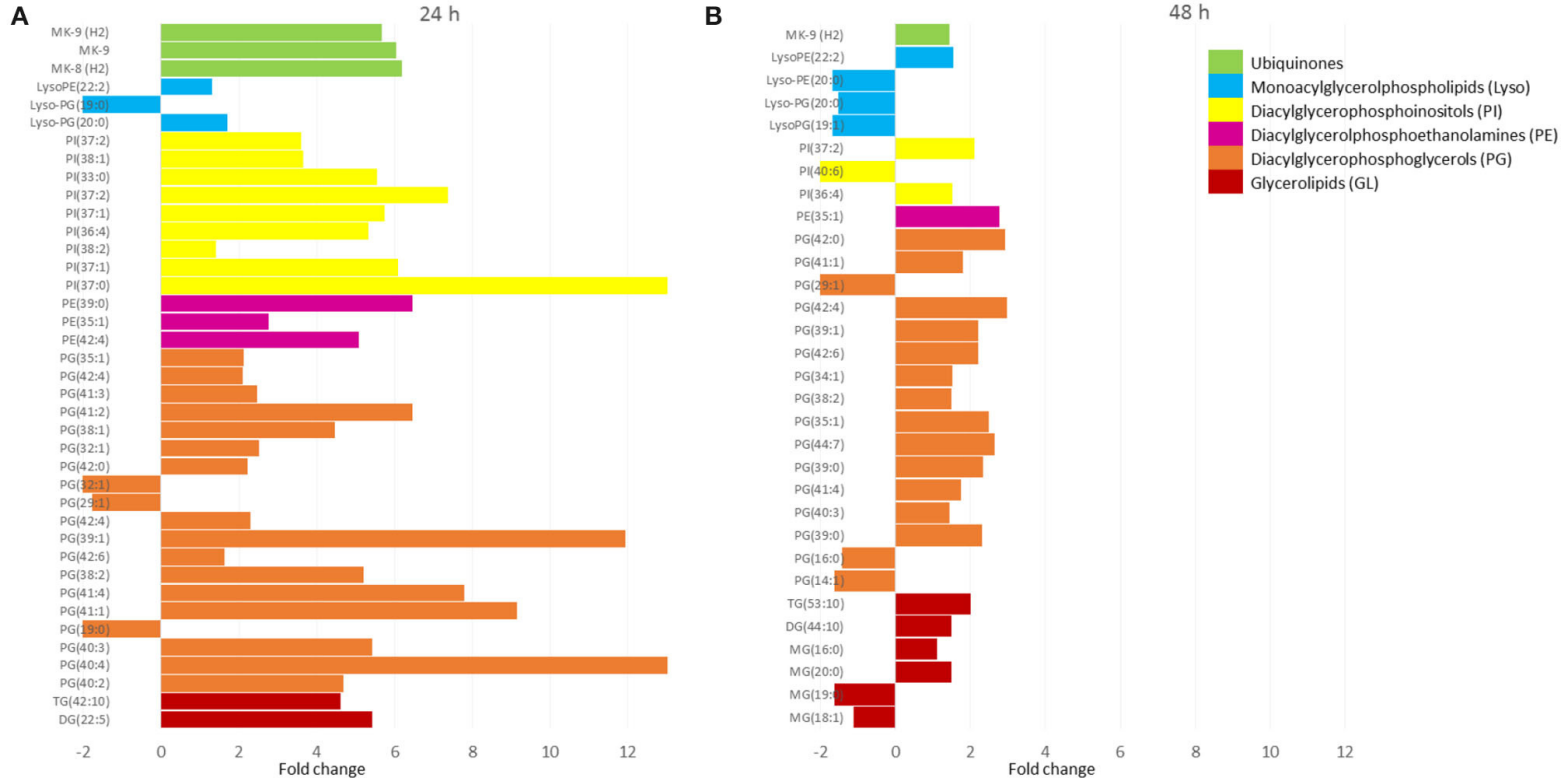
The lipid profiles of bacteria after 24 h **(A)** and 48 h **(B)** exposure to propolis. The m/z values of features were assigned to singly protonated ions (M+H)+ or (M+Na)+ with an allowed 0.05 m/z mass difference. Only statistically significantly changed features were annotated. The molecular features present in propolis extract were removed from test samples. X-axis values correspond to the fold change observed for annotated lipids, which were grouped in classes (different colors). Values above zero represent upregulation, while below zero downregulation. MG, monoacylglycerols; DG, diacylglycerols; TG, triacylglycerols; PG, diacylglycerophosphoglycerols; PE, diacylglycerophosphoethanolamines; PI, diacylglycerophosphoinositols; Lyso-GP, monoacylglycerophosphoglycerols; Lyso-PE, monoacylglycerolphosphoethanolamines; MK, menaquinones.

Because a large part of mycobacteria DNA coding capacity is used for the production of enzymes that are involved in lipogenesis and lipolysis (Cole, 1998) lipids constitute up to 40% of the dry weight of the tubercle bacillus (Chiaradia et al., 2017). Thick and specific mycobacterial cell envelope composed of rich lipid fraction is considered among the main reasons for these bacteria resistance (Chiaradia et al., 2017). Taking into account, the importance of lipids in mycobacteria, we analyzed their apolar and amphipathic fractions. The PCA scores revealed no differences between hydrophobic extracts (apolar lipids) obtained from treated and control bacteria (Figures 1A,B). The visible separation between test groups was obtained for methanolic-aqueous extracts after 24 h (Figure 1C). The separation was still maintained after 48 h, however, the sets were less aggregated (Figure 1D). Whole cells chloroform–methanolic extracts were not analyzed further due to the absence of any statistical significance. The metabolites present in other extracts were annotated only if the difference between the tested and control groups was statistically significant (p < 0.05). Small metabolites were subjected to enrichment analysis (Figure 2), while lipids (amphipathic lipids) profiles were presented in Figure 3.

Bacteria subjected to propolis exposure revealed the enrichment in several metabolic pathways, however, the number of altered pathways was higher in the first 24 h (Figure 2). Phenylanaline, tyrosine, and tryptophan metabolism, pantothenate and CoA biosynthesis, and riboflavin metabolism were the main altered in the first 24 h. The following 24 h of exposure resulted in the highest enrichment in nicotinate and nicotinamide metabolism, pantothenate and CoA biosynthesis, cysteine and methionine metabolism, or valine, leucine, and isoleucine metabolism. The metabolic activity related to sugars, amino-sugar and nucleotide sugar pathways, inositol phosphate pathway, pentose phosphate pathway, and glycolysis/gluconeogenesis was observed only in the first 24 h of exposure (Figure 2).

The methanolic–aquous extracts contained a small portion of lipids that belonged to glycerolipids (GL), glycerophospholipids (PG, PE, PI, and Lyso), and prenol lipids (ubiqinones) (Figure 3). The first 24 h of exposure to propolis resulted in upregulation of detected compounds from 2 to more than 12 times (with few exceptions). After the next 24 h, the fold change decreased significantly and did not exceed 3 in relation to control samples. Glicerophospholipids dominated with the most abundant diacylglycerophosphoglycerls (PGs), followed by diacylglycerophosphoinositls (PIs), diacylglycerophosphoethanolamines (PEs), and their lyso forms (Lyso).

### Expression of Genes Coding Sigma Factors

**Figure 4:**
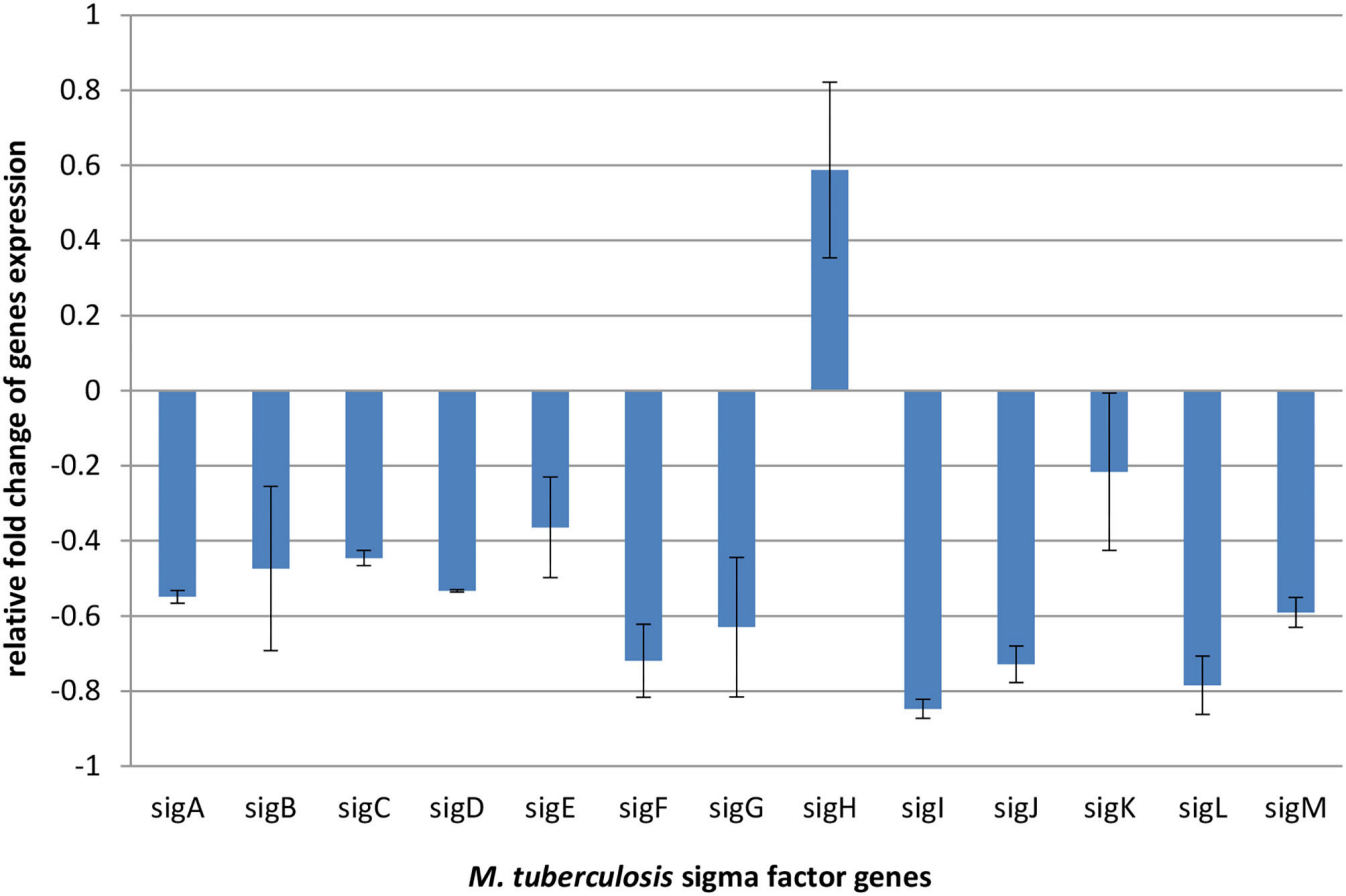
Relative fold change expression of *M. tuberculosis* H37Ra sigma factors genes after 24 h of exposure to an effective dose (256 μl/ml) of propolis extract, normalized to 16SmRNA (control).

Sigma factors play general regulatory functions related to gene expression, transcription, and translation. They are also needed in fatty acids and lipid metabolism, in response to antibiotics, xenobiotic stimuli, and oxidative stress, and also play role in virulence and under starvation conditions (Chauhan et al., 2016). Changes in RNA polymerase–sigma factors interaction leads to initiation of transcription of respective gene sets and consequently influences the particular metabolic pathways (Chauhan et al., 2016). Therefore, the evaluation of expression levels of sigma factors provides a global view on the bacterial response to compounds inhibiting their growth. To better explain the observed metabolomic changes in mycobacteria under exposure to propolis, we performed transcriptomic analysis, which revealed shifts in the sigma factors regulatory network. The observed downregulation of all sigma factors except sigH is presented in Figure 4.

## Discussion

In this work, we made a first attempt to determine mechanism of action of Nepalese propolis extract against *Mycobacterium tuberculosis*. The bacteria susceptibility evaluation revealed that MIC values determined for antibiotics were in general agreement with literature data for *M. tuberculosis* H37Ra strain (Heinrichs et al., 2018). A better activity was observed for *Trigona* sp. propolis extract comparing to *Apis mellifera* L. propolis extract. The previous phytochemical analysis showed that both extracts contain 2'-hydroxyformononetin, odoratin, vestitol, butein, dalbergin, 7-hydroxyflavanone, and pinocembrin (Okińczyc et al., 2020), however, their content may differ. Bees selectively collect plant resins and usually prefer material from only some plant species in this same area (Simone-Finstrom and Spivak, 2010). In addition, bees mix plant resins with their saliva increasing the variability of the final product (Simone-Finstrom and Spivak, 2010). The previously reported antibacterial activity of propolis was mainly investigated for its poplar type (Cushnie and Lamb, 2005; Almuhayawi, 2020), while Nepalese propolis is a subtype of “red propolis” and the chemical composition of this type is strongly different from poplar propolis (Okińczyc et al., 2020). Therefore, the metabolomic analysis was performed for more active *Trigona* sp. propolis extract to determine if its antimycobacterial activity is similar to activity of poplar propolis against other bacteria.

As we previously investigated, DMSO causes minor metabolic changes in the bacterial cells influencing the composition of mycobacterial membrane lipids. The noticed changes in lipid profiles may be a consequence of the direct physico-chemical interactions between DMSO and lipid bilayers (Sieniawska et al., 2020). DMSO competes with the lipid head group for favorable interactions with water, decreasing the head group hydrated volume (Schrader et al., 2016), and as a result the desorption of lipids from the bilayer occurs. For this reason, if DMSO is used as a solvent-solubilizing active compounds, its influence on the bacterial lipid profile should not be neglected. Therefore, in this experiment, the control cultures were supplemented with 2% DMSO to make a baseline for observed changes.

The detailed metabolomic and transcriptomic analysis performed in this study indicated target points in bacterial cells under propolis extract influence. The profile of lipids forming outer and middle layers of the mycobacterial cell envelope was not changed by bacteria exposure to propolis (Figure 1). However, some fluctuations in the profiles of amphipathic glycerophospholipids were observed (Figure 3). Glycerophospholipids are the largest class of mycobacterial lipids (Crellin et al., 2013). In comparison to control samples, bacteria exposed to propolis produced higher amounts of molecules that are major structural components of the mycobacterial plasma membrane: diacylglycerolphosphoethanolamines (PEs), diacylglycerophosphoinositols (PIs); and precursors for lipomannan and lipoarabinomannan (PI) (Crellin et al., 2013). The several-fold increase of these lipids was the early (24 h) bacterial response to the stressing agent. Bacteria sealed/repaired the cell membrane, indicating some destabilization caused by exposure to propolis. At the same time, a significant increase in the number of mycobacterial ubiquinones was observed (Figure 3A). Menaquinones (MK) are placed in the cell membrane and are needed for ATP generation through the proton flow. They accept electrons from hydrogenases, dehydrogenases, or oxidoreductases, and transfer them to the terminal oxidases in aerobic respiration or terminal reductases in anaerobic respiration (Upadhyay et al., 2015). The MK upregulation may suggest the increased demand for energy during the first day of exposure. After the next 24 h, the profile of detected lipids changed significantly (Figure 3B). The upregulation was still visible, however, the level of changes was much lower. This observation may indicate that bacteria slowly restored the balance at the cell membrane level.

The enrichment analysis revealed bacterial metabolic pathways affected by exposure to *Trigona* sp. propolis. The early metabolic response involved more pathways than observed after 48 h of incubation, however, the highest enrichment ratio was observed after 48 h, indicating long-lasting influence of propolis. Also, the significance of the pathways was changed over time (Figure 2). In the first 24 h of exposure bacteria intensified Coenzyme A (CoA) biosynthesis. CoA is a common acyl carrier in numerous enzymatic reactions central to intermediary metabolism, including key intermediates in energy production (Karp et al., 2019). This observation correlated with detected menaquinones upregulation and confirmed the increased energy demand. Also, the riboflavin metabolism was affected in the first 24 h. Riboflavin is the precursor for the essential flavin cofactors, flavin mononucleotide (FMN), and flavin adenine dinucleotide (FAD) working with flavoproteins (Monteira, 2013). FMN and FAD catalyze different redox reactions by transferring either one or two electrons, hydrogen atoms, or hydronium ions (Monteira, 2013). Flavoproteins are involved in numerous metabolic pathways, including electron transport, synthesis of CoA, catabolism of amino acids (leucine, isoleucine, valine, and lysine), beta-oxidation of fatty acids, DNA repair, or nucleotide biosynthesis (Mansoorabadi et al., 2007). Other enriched metabolic routes involved glycolysis/gluconeogenesis needed for energy production, but also pentose phosphate pathway, which produces 5-carbon sugars for the biosynthesis of DNA and RNA, 4-carbon sugar for the biosynthesis of aromatic amino acids (phenylalanine, tyrosine, and tryptophan), or 7-carbon sugar for the biosynthesis of lipid A (Karp et al., 2019). The pathways related to metabolism of sugars (mannose and galactose), amino-sugar and nucleotide sugar or inositol phosphate pathway confirm that bacteria intensified the essential metabolic processes to overcome the action of stressing agent.

After the second day of bacteria exposure to propolis, nicotinate and nicotinamide metabolisms had the highest enrichment ratio and significance (Figure 2B). Nicotinate is a basic structure of nicotinamide adenine dinucleotide (NAD) and nicotinamide adenine dinucleotide phosphate (NADP), which are two of the most essential coenzymes in redox reactions in the cell (Karp et al., 2019). The high enrichment of this pathway may indicate increased redox processes that are vital in all parts of metabolism, including energy generation. An additional observation was the intensification of amino acids metabolism, especially sulfur containing cysteine and methionine. Sulfur metabolism in mycobacteria is crucial for the maintenance of redox homeostasis. Cysteine-derived cysteamine moiety is a part of low molecular mass thiol (1-d-myo-inosityl-2-(N-acetyl-cysteinyl) amino-2-deoxy-α-d-glucopyranoside, and mycothiol) unique to these organisms (Karp et al., 2019). Mycothiol is required to detoxify-alkylating agents, reactive oxygen and nitrogen species, and antibiotics. It provides a highly reducing environment within the cell (Karp et al., 2019). The other amino acid, methionine, is mainly consumed for the production of S-adenosyl-L-methionine (SAM), the primary methyl donor in the cell. SAM also participates in the formation of Factor 420 (a redox-active compound) and mycofactocin (a redox electron carrier) (Karp et al., 2019). What is more, methionine spontaneously can react with hydrogen peroxide to scavenge it without any enzyme required (Karp et al., 2019). Such information may indicate that on the second day of incubation bacteria increased metabolic activity related to redox balance. These results corroborate with the previously described pro-oxidant activity of Nepalese propolis in *Candida albicans*, for which production of the superoxide anion radical (O2^•−^) and hydroxyl radical (OH^•^) was observed under exposure to propolis (Okińczyc et al., 2020).

In addition, the transcriptomic analysis revealed shifts in the sigma factors regulatory network under exposure to propolis. Accessory sigma factors direct RNA polymerase to transcribe specific gene sets and activate bacterial adaptive responses to environmental stimuli. Chauhan et al. demonstrated that 13 sigma factors create network hierarchy and five communities, coordinately responding to the different stimuli. The network consists of three levels. Master regulators SigA, SigB, SigH, and SigM, act downstream through the middle (SigE, SigG, SigL, SigJ, and SigF) and lower (SigD, SigK, SigI, and SigC) levels. With anti-sigma, anti-anti-sigma, and transcription factors, sigma factors create a complex system regulating mycobacterial gene expression (Chauhan et al., 2016). Almost all the components of this network were slightly downregulated under propolis influence. Overexpression was noticed only for *sigH*, which belongs to extra-cytoplasmic function (ECF) proteins regulating bacterial interactions with the extracellular environment and stress adaptation. ECF sigma factors are held in an inactive form by a cognate anti-sigma factor until released (Manganelli et al., 2002). SigH activity is regulated post-translationally by its anti-sigma factor, RshA, which senses stresses and releases SigH to bind to core RNA polymerase and activate transcription of its regulon. Genes directly regulated by SigH form a group of about 25 genes. These genes indicate that in addition to regulation of redox homeostasis and protein turnover, SigH regulates genes required for repair of DNA damage, recovery of ribosome function and translation, sulfur transport, and synthesis and salvage of sulfur-containing amino acids (Sharp et al., 2016). Observed *sigH* overexpression is consisted with changes in the bacterial metabolic pathways intensified on the second day of exposure (Figure 2), confirming that bacteria counteracted the action of propolis by the intensification of redox reactions and metabolism of sulfur containing amino acids. The demand for *sigH* transcripts indicates long-lasting stress related to the tested propolis extract.

The previously performed phytochemical analysis of propolis 70% ethanolic extract used in this work confirmed the presence of flavanones (pinocembrin and 7-hydroxyflavanone), isoflavones (2'-Hydroxyformononetin and liquiritigenin), chalcones (butein), neoflavanoids (dalbergione), and their quinone derivates (2-(1-Phenylprop-2-enyl)benzene-1,4-diol) (Okińczyc et al., 2020). The literature data indicate the multifactorial antibacterial mechanisms of action attributed to flavonoids, including inhibition of nucleic acid synthesis, disruption of cell-membrane functions and bacterial energy metabolism, or prevention of bacterial adhesion and biofilm formation (Cushnie and Lamb, 2005). Determined in this study, antimycobacterial activity of propolis extract is in agreement with action reported for individual flavonoids, targeting cell membrane, energy metabolism, and additionally redox homeostasis.

## Conclusion

In this work, we observed metabolic and transcriptomic changes in mycobacterial cells induced by the influence of the extract of Nepalese propolis produced by *Trigona* sp. The early bacterial response was correlated with metabolic pathways involved in the energy production. Increased demand for energy was confirmed by higher levels of menaquinones and may be linked with synthesis of cell membrane molecules observed in increased amounts after first day of exposure. The membrane damage may be a result of accumulated free radicals. After the second day, bacteria restored membrane balance, however, the metabolic pathways related to redox homeostasis were significantly influenced. Increase of sulfur-based molecules, which specifically participate in the neutralization of different kinds of radicals, indicate that bacteria challenged the highly oxidizing environment within the cell. Transcriptomic analysis confirmed that SigH accessory factor induced the mechanisms related to the neutralization of free radicals in the cells. The upregulated expression of sigH was observed after 24 h, however, at the metabolic level, this activity was pronounced on the second day of exposure, indicating the prolonged influence of propolis flavonoids on bacteria. The performed experiment proves for the first time the successful application of LC–MS metabolomics combined with targeted transcriptomics in the determination of the mode of action of Nepalese propolis extract.

## Data Availability Statement

The original contributions presented in the study are included in the article/supplementary material, further inquiries can be directed to the corresponding author/s.

## Author Contributions

RS and ES: conceptualization, methodology, validation, writing—original draft preparation, and visualization. ES: software, supervision, and project administration. JW, RS, WT, ES, PO, and JG: investigation. RS, ES, JW, and PO: writing—review and editing. All authors have read and agreed to the published version of the manuscript.

## Conflict of Interest

The authors declare that the research was conducted in the absence of any commercial or financial relationships that could be construed as a potential conflict of interest.

## Publisher's Note

All claims expressed in this article are solely those of the authors and do not necessarily represent those of their affiliated organizations, or those of the publisher, the editors and the reviewers. Any product that may be evaluated in this article, or claim that may be made by its manufacturer, is not guaranteed or endorsed by the publisher.
